# The Central Facial Defect Reconstruction Using a Radial Forearm Free Flap after Malignant Cutaneous Tumor Ablation

**DOI:** 10.3390/jcm12227148

**Published:** 2023-11-17

**Authors:** Kyusang Cho, Jinsol Park, Seokchan Eun

**Affiliations:** Department of Plastic and Reconstructive Surgery, Seoul National University College of Medicine, Seoul National University Bundang Hospital, Seongnam 13620, Republic of Korea; whrbtkd0912@naver.com (K.C.); fine5216@naver.com (J.P.)

**Keywords:** radial forearm, free flap, facial reconstruction, oncologic defect

## Abstract

Purpose: Acquired defects of the central face pose significant challenges in achieving acceptable cosmetic and functional outcomes. The site, size, and depth of tissue loss often render local tissues inadequate for the repair of major nasal defects. In this article, we aim to demonstrate the efficacy of radial forearm-free flaps as an ideal choice for various central facial unit reconstructions. Methods: This study encompassed patients treated between 2020 and 2022 who underwent facial reconstruction using radial forearm flaps. These flaps were employed in eleven patients with defects involving the lower lid, nose, upper lip, and lower lip. Additionally, we used osteocutaneous flaps in one patient to reconstruct a right nasal bone defect. In three patients requiring medial and lateral canthal tendon reconstruction in one case and oral sphincter reconstruction in two cases, the palmaris longus tendon was included with the flap. Results: In the majority of cases, we achieved good to excellent aesthetic and functional results. Notably, there were no instances of flap failure or partial necrosis in this series. All patients experienced uneventful healing at the donor site. Conclusions: The radial forearm-free flap stands as an ideal and reliable method for reconstructing various facial defects. It offers efficient and thin-conforming skin coverage.

## 1. Introduction

The central face, comprising vital functional elements such as the eyes, nose, and mouth, presents a unique and intricate challenge for reconstruction. Tissue loss, whether in terms of site, size, or depth, can often make local tissues unsuitable or insufficient [1]. The face is divided into facial aesthetic subunits, each characterized by unique skin and soft tissue thicknesses and tissue characteristics [2,3,4]. However, the restoration of facial aesthetic subunits can be particularly challenging, especially when extensive defect excisions are necessary, often due to the limited availability of local tissues. Free tissue transfer is a particularly valuable approach, offering robust healing capabilities after a single surgery and saving precious time [5]. Given its numerous advantages, the radial forearm-free flap plays a pivotal role in the reconstruction of various facial components [6,7,8]. The pliability of the flap allows for its folding without compromising vascular supply, enabling the reconstruction of complex three-dimensional structures, such as nasal penetration defects or full-thickness defects of the lips [9]. By including one or more skin islands with the flap and reestablishing sensory function through anastomosis of the lateral antebrachial cutaneous nerve with a sensory recipient nerve, this article exemplifies the adaptability and reliability of the radial forearm flap in 11 patients undergoing facial reconstructions following cutaneous cancer ablation surgery.

## 2. Materials and Methods

Between January 2020 and December 2022, we treated 11 patients with different facial defects resulting from tumor ablation. These patients underwent immediate microsurgical reconstruction employing radial forearm-free flaps. The flaps were utilized in cases of lower lid defects (two cases), nasal defects (four cases), upper lip defects (three cases), and lower lip defects (two cases). Tumor types included two cases of basal cell carcinoma, four of squamous cell carcinoma, three of malignant melanoma, and two of Merkel cell carcinoma. All flaps were designed distally on the forearm, encompassing the radial bone, radial artery, and cephalic vein. The skin island extended above the fascia, reaching down to the palmaris longus and the brachioradialis tendon to prevent tendon exposure. In one patient requiring reconstruction of the right nasal bone defect, an osteocutaneous flap was employed, including a 3 cm radius segment. Three patients required the inclusion of the palmaris longus tendon, serving for medial and lateral canthal tendon reconstruction in one case and oral sphincter reconstruction in two cases. Flap inset at the recipient site and vascular anastomoses were performed in an end-to-end fashion. Donor site defects were grafted with full-thickness skin from the groin area for all patients. Forearm splinting was maintained for two weeks with a short arm splint and extended to four weeks for cases involving osteocutaneous flap harvesting.

## 3. Results

All 11 patients achieved successful reconstruction with good to excellent aesthetic and functional outcomes, in the patient’s own words. There were no instances of flap failure or partial necrosis within this series (Table 1). Each patient presented unique challenges regarding the reconstructed sites, recipient vessel choices, flap inset, and minor revisions. Approximately two-thirds of patients underwent minor revisions for flap bulkiness several months after the primary surgery. Notably, patients with lower eyelid reconstruction displayed satisfactory results without scleral show or mild lid lag. Patients undergoing nasal wall reconstruction experienced mild nasal stuffiness due to flap bulkiness, prompting the use of nasal retainers for one month post-surgery to ensure nasal valve patency. Patients with oral sphincter reconstruction demonstrated good oral continence, albeit with temporary mild drooling in the early post-operative period, especially among those undergoing lower lip reconstruction. All patients experienced uneventful healing at the donor site, with the exception of one patient who encountered partial skin graft failure.

### 3.1. Patient Reports

#### 3.1.1. Patient I

**Figure 1:**
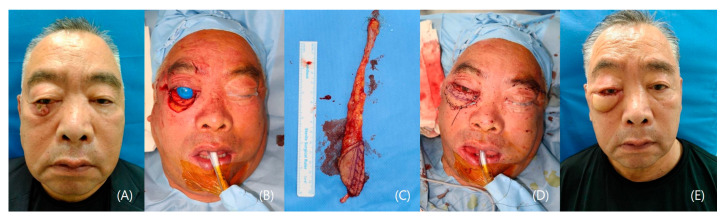
A case of a lower eyelid squamous cell carcinoma patient. (**A**) Preoperative photo. (**B**) Defect after wide excision of the tumor. (**C**) Radial forearm flap, including the palmaris longus tendon. (**D**) Immediate post-operative photo. (**E**) Post-operative 1-month photo.

A 65-year-old male patient underwent a biopsy, confirming squamous cell carcinoma on his right lower eyelid. General anesthesia was administered, followed by a wide excision with a 10 mm safety margin. The resulting defect measured 6 × 4 cm, fully exposing the eyeball. A 6 × 4 cm radial forearm flap, including the palmaris longus tendon, was harvested from the right forearm. An end-to-end anastomosis connected the flap to the superficial temporal vessels, while the palmaris longus tendon reconstructed the lower tarsal plate. Additionally, buccal mucosa was harvested and placed on the conjunctival aspect of the flap. Post-operation, a pleasing lower eyelid was reconstructed both aesthetically and functionally. The patient did not suffer any scleral show, dry eye, or other eye symptoms. The patient expressed satisfaction with the functional and aesthetic results, with minimal donor site morbidity (Figure 1).

#### 3.1.2. Patient II

**Figure 2:**
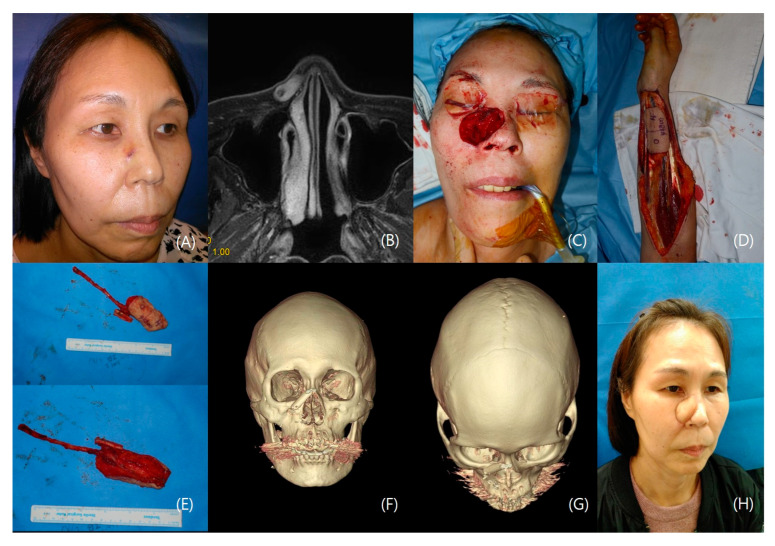
A case of a patient with basal cell carcinoma in the right nose. (**A**) Preoperative photo. (**B**) Pre-operative MRI (**C**) 5 × 4 cm defect including right nasal bone and nasal mucosal lining. (**D**) Design of the radial forearm flap, including the nasal mucosal lining (**E**) Radial forearm osteocutaneous flap harvested. (Distal radius included) (**F**) Post-operative 3D-CT AP view. (**G**) Post-operative 3D-CT Bird’s eye view. (**H**) Postoperative 5-month photo.

A 56-year-old female patient underwent a biopsy confirming basal cell carcinoma on her right nose, with the mass involving the right nasal bone. After administering general anesthesia, a wide excision with a 10 mm safety margin was performed, resulting in a through-and-through defect measuring 3.5 × 3 cm, including the right nasal bone and nasal inner mucosa. A 5 × 4 cm radial forearm flap, including the distal radius bone, was harvested. The distal flap was folded inside to reconstruct the nasal mucosal lining. The distal radius bone was fixed with a microplate and microscrews to reconstruct the lateral nasal bone segment, ensuring successful reconstruction of the nasal airway. Post-operation, the patient achieved both functional and aesthetic results with oncological safety (Figure 2).

#### 3.1.3. Patient III

**Figure 3:**
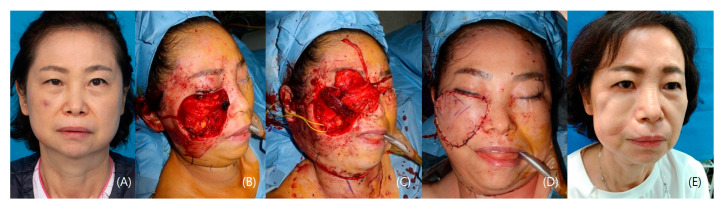
A case of a patient with Merkel cell carcinoma in the right cheek. (**A**) Preoperative photo. (**B**) 7 × 7 cm defect in the right cheek. (**C**) Radial forearm flap inserted for facial reanimation (Anterior interosseous nerve to Zygomatic branch of the facial nerve, Pronator quadratus for the zygomaticus major muscle) (**D**) Immediate post-operative photo. (**E**) 10-month post-operative photo.

A 59-year-old female patient underwent a biopsy, confirming Merkel cell carcinoma on her right cheek. Following general anesthesia, a wide excision with a 25 mm safety margin resulted in a defect measuring 7 × 7 cm, including the periosteum. A 7 × 7 cm radial forearm flap was harvested from the right forearm, encompassing the pronator quadratus muscle and innervating anterior interosseous nerve. An end-to-end anastomosis connected the flap to the facial artery and a branch of the external jugular vein. To reconstruct the zygomaticus major muscle, the pronator quadratus muscle was anchored to the orbicularis oris muscle and the zygomatic arch periosteum. Neurorrhaphy connects the zygomatic branch of the facial nerve with the anterior interosseous nerve (AIN). After a 5-month follow-up, the patient’s facial animation was recovering, and the patient was satisfied with the aesthetic result (Figure 3).

#### 3.1.4. Patient IV

**Figure 4:**
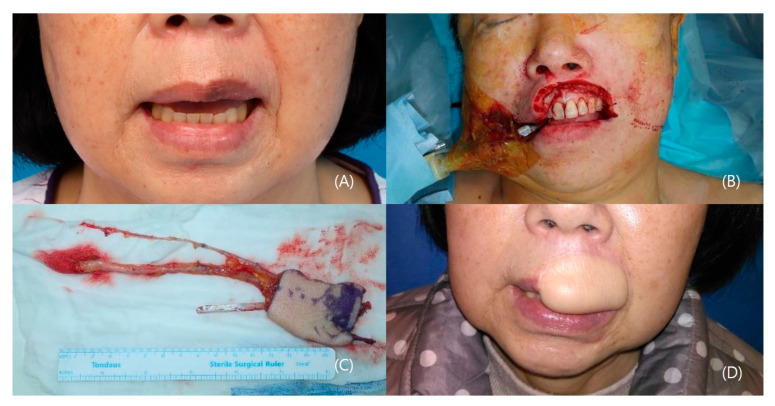
A case of an upper lip malignant melanoma patient. (**A**) Preoperative photo. (**B**) 6 × 5 cm defect after wide excision. (**C**) Radial forearm tendinocutaneous flap (Palmaris longus tendon included). (**D**) 2 years post-operative photo.

A 62-year-old female patient underwent a biopsy, confirming melanoma on her upper lip. General anesthesia was administered, followed by a wide excision with a 20 mm safety margin, resulting in a defect measuring 3 × 5 cm and fully exposing the gingiva and teeth. A 6 × 5 cm radial forearm flap was harvested from the right forearm, including the palmaris longus tendon. An end-to-end anastomosis connected the flap to the facial artery, vein, and a branch of the external jugular vein. The palmaris longus tendon was secured to the modiolus. Post-operation, the patient achieved oncological safety with functional outcomes. The patient was able to pronounce normal sentences without difficulty and also drink water without drooling. The aesthetic outcome was acceptable (Figure 4).

#### 3.1.5. Patient V

**Figure 5:**
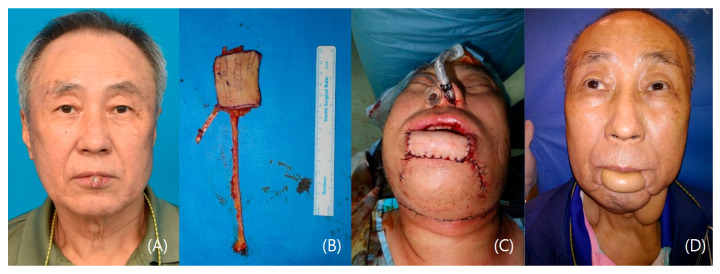
A case of a lower lip squamous cell carcinoma patient. (**A**) Preoperative photo. (**B**) 6 × 4 cm radial forearm flap (Palmaris longus included) (**C**) Immediate post-operative photo. (**D**) 10-month post-operative photo.

A 70-year-old male patient underwent a biopsy, confirming squamous cell carcinoma on his lower lip. After general anesthesia, a wide excision with a 10 mm safety margin was performed, resulting in a defect measuring 3 × 4 cm and fully exposing the gingiva and teeth. A 6 × 4 cm radial forearm flap was harvested from the right forearm, including the palmaris longus tendon. An end-to-end anastomosis connected the flap to the facial vessels. The palmaris longus tendon was secured to the modiolus. The patient achieved oncological safety and did not suffer any complications such as drooling, dry mouth, or any others (Figure 5).

## 4. Discussion

Reconstructing defects in the central face, encompassing vital anatomic structures such as the eyelid, nose, upper lip, lower lip, and cheek skin, remains a formidable challenge [8,10]. Numerous previous attempts have been made to restore facial subunits using locoregional flaps. However, these efforts have encountered limitations, particularly in the context of central face reconstruction, which demands the restoration of its distinctive aesthetic and functional characteristics. Multistage local or regional flap approaches involve multiple surgeries with time delays, making them less attractive. Free tissue transfer stands as an ideal approach for facial subunit oncologic defect reconstruction, as it allows patients to continue their therapies without the need for multiple surgeries [10,11]. In this study, all patients underwent immediate free flap reconstruction after cancer surgery.

Since its introduction as a free flap, the radial forearm flap has undergone various design modifications for the reconstruction of diverse defects. Key features, such as the minimal bulk and pliability of the skin island, make this flap an ideal choice [12,13]. The flap’s pedicle length is among the largest that can be obtained from any free flap. Both the radial artery and cephalic vein boast substantial diameters, providing reliable options for anastomoses [14,15,16]. Moreover, concomitant veins can also be reliably used for venous anastomosis [17]. Various composite flaps have been described, including fasciocutaneous, adipofascial, osteocutaneous, and tendinocutaneous flaps, designed and transferred from the radial forearm based on the defect size, location, and tissue requirements [18,19,20].

Immediate reconstruction using a tendinocutaneous radial forearm flap proves highly effective for upper and lower eyelid reconstruction. Securing the palmaris longus tendon to the medial and lateral orbital rim periosteum and the deep temporal fascia can prevent lower lid retraction, which is aesthetically undesirable [21,22]. Grafting the buccal mucosa reconstructs the inner conjunctival layer. The pedicle vessels are consistently long enough for anastomosis to the superficial temporal artery and vein. 

The osteocutaneous radial forearm flap proves highly useful for patients with nasal composite defects, which can be emotionally distressing, particularly for young women. Local or regional flap reconstruction for such complex defects has been considered exceedingly difficult due to the location and extent of the defect [10,11]. In our opinion, a one-stage reconstruction of the external skin, nasal skeleton, and mucosa using a free flap offers an optimal solution. The bone segment should be harvested according to the length and height of the nasal skeleton defect. Shaping the radius segment using a burr results in a stable bone graft that can be easily fixed with a single screw. Otherwise, achieving rigid fixation with titanium miniplates, especially in the distal nasal skeleton, can be challenging [8,18,20]. While considering the restoration of the nasal framework with cartilage, it was crucial to prioritize the need for a rigid framework to prevent airway collapse. Consequently, a single-stage reconstruction using the radius bone was chosen as the preferred approach. The forearm’s soft tissue can provide an inner lining to the nose, orbit, or sinuses in the form of skin or fascia and an outer lining of the same structures using single or multiple skin paddles. Nasal airway patency presents a challenge for nasal composite reconstructive procedures. This challenge is most often due to nasal valve collapse resulting from the loss of the bony and cartilaginous framework [11,20]. While flap soft-tissue collapse and skin shrinkage may occur later, revision surgeries were performed in attempts to improve the nasal airway and refine the soft tissue of the nose, if necessary.

Restoring the shape of the lip is a challenging task, given its distinctive aesthetic and functional characteristics, including the vermillion, mucosal surface, and orbicularis oris muscle. However, the use of locoregional flap reconstruction in this area can lead to the development of microstomia, particularly after cancer ablation, making the use of free flaps the preferred choice [23].

The palmaris longus tendon, along with a skin island, is also valuable for reconstructing the upper lip while maintaining adequate oral continence. Anchoring the palmaris longus tendon to both sides of the modiolus and orbicularis oris muscle ensures adequate lip closure and favorable aesthetic outcomes [24,25]. The pedicles are sufficiently long for anastomosis with the facial or superficial thyroid vessels. A common criticism of the forearm donor site pertains to the appearance of the skin graft. Our approach involves suprafascial dissection, preserving the integrity of the fascia, and protecting all tendons with their paratenon, thereby maximizing the success of the full-thickness skin graft while minimizing contracture, tendon exposure, and morbidity. Dermal substitute grafts are performed concomitantly to achieve stable cosmetic results [26].

In summary, we demonstrate the viability of the radial forearm-free flap as an initial reconstruction method after central face cancer surgery. General satisfaction with cosmesis can be achieved, accompanied by a good success rate in flap survival in this series.

## Figures and Tables

**Table 1:** Patient demographics and surgical details.

Patient No.	Sex/Age	Defect Location	Type of Tumor	Extra Flap Composition	Flap Dimension (cm^2^)	Complications/Outcomes
1	M/78	Lower eyelid	MM	PL	8 × 5	Mild lid-lag
2	M/65	Lower eyelid	SCC	PL	6 × 4	Mild lid-lag
3	F/51	Nasal wall	SCC	None	5 × 4	None
4	F/65	Nasal wall	SCC	None	4 × 3	None
5	F/56	Nasal wall	BCC	DR	5 × 4	None
6	M/76	Ala	BCC	None	7 × 5	None
7	F/58	Cheek	MCC	PQ, AIN	7 × 7	None
8	F/66	Cheek	MCC	None	6 × 5	None
9	F/60	Upper lip	MM	PL	6 × 5	None
10	F/58	Upper lip	SCC	PL	5 × 3	None
11	M/69	Lower lip	SCC	PL	6 × 4	Mild drooling

MM, Malignant melanoma; SCC, Squamous cell carcinoma; BCC, Basal cell carcinoma; MCC, Merkel cell carcinoma; PL, Palmaris longus tendon; DR, Distal radius bone; PQ, Pronator quadratus muscle; AIN, Anterior interosseous nerve.

## Data Availability

The data presented in this study are available on request from the corresponding author.
